# Porcine Circovirus Type 3 (PCV3) in Poland: Prevalence in Wild Boar Population in Connection with African Swine Fever (ASF)

**DOI:** 10.3390/v16050754

**Published:** 2024-05-10

**Authors:** Maciej Piotr Frant, Natalia Mazur-Panasiuk, Anna Gal-Cisoń, Łukasz Bocian, Magdalena Łyjak, Anna Szczotka-Bochniarz

**Affiliations:** 1 Department of Swine Diseases, National Veterinary Research Institute, Partyzantów Avenue 57, 24-100 Puławy, Poland; anna.gal@piwet.pulawy.pl (A.G.-C.); magdalena.lyjak@piwet.pulawy.pl (M.Ł.); anna.szczotka@piwet.pulawy.pl (A.S.-B.); 2 Virogenetics Laboratory of Virology, Małopolska Centre of Biotechnology, Jagiellonian University, Gronostajowa 7A, 30-387 Kraków, Poland; natalia.mazur-panasiuk@uj.edu.pl; 3 Department of Epidemiology and Risk Assessment, National Veterinary Research Institute, Partyzantów Avenue 57, 24-100 Puławy, Poland; lukasz.bocian@piwet.pulawy.pl; 4 Department of Cattle and Sheep Diseases, National Veterinary Research Institute, Partyzantów Avenue 57, 24-100 Puławy, Poland

**Keywords:** ASF, ASFV, PCV3, PCV4, wild boar

## Abstract

Human health is dependent on food safety and, therefore, on the health of farm animals. One of the most significant threats in regard to swine diseases is African swine fever (ASF). Infections caused by porcine circoviruses (PCVs) represent another important swine disease. Due to the ubiquitous nature of PCV2, it is not surprising that this virus has been detected in ASFV-affected pigs. However, recent data indicate that coinfection of PCV3 and ASFV also occurs. It is still unclear whether PCV infection plays a role in ASFV infection, and that subject requires further analysis. The aim of this study was to assess whether PCV3 and PCV4 are present in the wild boar population in Poland (real-time PCR). The analysis was performed on wild boar samples collected for routine ASF surveillance in Poland, between 2018 and 2021. By extension, the obtained data were compared in regard to ASFV presence in these samples, thus investigating the odds of ASFV infection on the grounds of the PCV carrier state in free-ranging Suidae in Poland. In addition, sequencing of PCV3 and phylogenetic analysis were performed, based on a full genome and a capsid gene. In the current study, we demonstrated the high prevalence of PCV3 in the wild boar population in Poland; meanwhile, PCV4 was not detected. The odds of ASFV infection on the grounds of the PCV3 carrier state in free-ranging Suidae in Poland was more than twice as high. Ten full genome sequences of PCV3 were obtained, all of them belonging to clade 3a. The similarity between them was in the range of 98.78–99.80%.

## 1. Introduction

Human health is dependent on food safety and, therefore, on the health of farm animals. Meat consumption in Poland in 2021 was around 74 kg/person/year [1], showing that this type of food is a highly popular element of our diet.

Since 2007, African swine fever (ASF) has presented the most serious threat to pig production in Eurasia. Since the hardly controllable wild boar population acts as the main reservoir of this infectious disease, the ASF virus (ASFV) poses the highest level of concern for food safety in Europe, including Poland. The ASFV is a large (170 to 193 kilobase pairs (kbp)) double-stranded DNA (dsDNA) virus, belonging to the *Asfarviridae* family. A total of 24 genotypes of the ASFV have been identified currently; however, only genotype II is present in Poland [2]. The disease has a morality rate of up to 100%; moreover, it is incurable and there is no commercially available vaccine approved for use in Europe (the trials of a commercial vaccine in Vietnam still require further analysis) [3]. Despite the fact that ASF is not a direct danger to human health, it does lead to enormous economic losses, causing the liquidation of farms and the placement of significant restrictions on trade and the pig market [3,4].

Infections caused by porcine circoviruses (PCVs) represent another important disease impacting Suidae. PCVs belong to the *Circoviridae* family and are small (approximately 2 kbp) single-stranded DNA (ssDNA) viruses [5,6]. Currently, four porcine circoviruses are known, namely PCV1, PCV2, PCV3, and PCV4. While PCV1 is harmless to pigs, PCV2 is common in pig farms and linked to several clinical manifestations, described as porcine circovirus-associated disease (PCVD, PCVAD) [7]. The clinical symptoms of PCV2 in pig farms are successfully controlled through the usage of commercial vaccines [5,8]. The presence of PCV2 has also been confirmed in wild boars in many countries, including Poland [9].

The situation is different for PCV3, which was discovered in 2015 (USA) [10], and PCV4, which was identified in 2019, in China [11]. Due to the fact that PCV3 and PCV4 were detected for the first time in the last few years, the knowledge about their pathogenicity is limited (mainly field observations). PCV3 infection may lead to inflammation and tissue injury in the host organism [12]; whereas, in the case of PCV4, porcine dermatitis nephropathy syndrome (PDNS), as well as respiratory or enteric diseases, and reproductive failures have been observed [13]. PCV3 seems to be widely spread throughout the pig population in Europe (i.e., in the United Kingdom, Italy, Sweden, Russia, Spain, Denmark, Germany, Hungary, Serbia) [14]. The virus has also been confirmed in the wild boar population in Spain [15], Germany [16], and Italy [17]. In Poland, PCV3 has been detected in domestic pigs [5]; however, there is no data on PCV3 in wild boars. 

To date, the presence of PCV4 has not been confirmed in Europe [13,18], and has not yet been investigated in Poland. The data regarding PCV3 and PCV4 infections in wild boars are scarce, as well as the clinical outcomes of infection and the long-term consequences for domestic pigs. There is no evidence whether transmission between pigs and wild boars is possible. 

Recent data indicate that coinfection with PCVs and ASFV is possible, at least in domestic pigs, which was confirmed with PCV2 in Mongolia [19], and with PCV3 in Mozambique [20] and Nigeria [21]. It is still unclear whether PCV2 or PCV3 infection predisposed the pigs to the ASFV infection, and that subject requires further analysis [19,20,21]. In both cases, the samples originated from domestic pigs, and there is still no data on ASFV coinfection with PCVs in wild boars. 

The aim of this study was to assess whether PCV3 and PCV4 are present in the wild boar population in Poland. The analysis was performed on wild boar samples collected for routine ASF surveillance in Poland, between 2018 and 2021. By extension, the obtained data were compared in regard to ASFV presence in these samples; thus, investigating the odds of ASFV infection on the grounds of the PCV carrier state in free-ranging Suidae in Poland.

## 2. Materials and Methods

### 2.1. Analyzed Biological Material

In total, 680 wild boar (found dead and road killed, or hunted) samples, collected between 2018 and 2021, were used for the analysis. Out of them, 520 were blood samples, and the remaining 160 were internal organs, which comprised of 78 spleens, 55 lungs, 13 kidneys, 9 lymph nodes, and 5 tonsils. The specimens were collected from 14 out of 16 Polish voivodships, thus covering almost the whole country; whereas, at the time, ASFV was present in 13 voivodships. The tested samples originated from a bank of samples on ASF, collected by the Polish National Reference Laboratory (NRL). The samples were collected by trained veterinary officers during the routine ASF monitoring program in Poland. The choice of sample location was determined by the availability of the biological material. Since ASFV is a virus of high concern, the whole laboratory analysis, beginning from the sample preparation through DNA extraction to molecular and serological studies, was conducted by qualified staff in a high-containment BSL-3 laboratory, located at the NRL in Poland. 

Based on the result of the ASFV screening, the samples for PCV3 and PCV4 analysis were further divided into two groups: originating from animals that had no contact with ASFV (group I; samples negative for ASF in a molecular and/or serological test) and originating from animals that had contact with the pathogen (group II; samples positive for ASF in molecular and/or serological test). There were 480 samples in group I and 200 in group II. Detailed data are shown in Table 1.

### 2.2. Detection of ASFV, PCV3, PCV4

#### 2.2.1. Sample Preparation

Specifically, 200 μL of 10% tissue homogenates (*v*/*w*, PBS) or whole blood were used for the extraction of the total DNA using a QIAamp DNA Mini Kit (Qiagen, Hilden, Germany), according to the manufacturer’s instructions. The DNA samples were stored at −20 °C, until further analysis [4].

#### 2.2.2. ASFV

The ASF status of the analyzed samples was confirmed with real-time PCR (for all samples) and the indirect immunoperoxidase technique (IPT) (for blood/serum samples).

The molecular detection of ASFV DNA was performed with a commercial kit, Virotype (Indical, Leipzig, Germany), according to the manufacturer’s instruction. The amplification process was conducted using one of two types of thermocyclers: Applied Biosystems 7500 (Applied Biosystems, Waltham, MA, USA) or QuantStudio™ 5 (Thermo Fisher Scientific, Waltham, MA, USA).

Prior to the serological analysis, the whole blood was centrifuged at 896–1514× *g* in order to obtain serum. The IPT working principle is similar to the ELISA test; however, the result is observed in a reverse-field microscope and there is no need for a plate reader. The IPT kit was provided by the European Union Reference Laboratory (EURL) for the ASF (CISA-INIA, Valdeolmos, Spain) and the analysis was conducted according to the EURL protocol.

#### 2.2.3. PCV3 and PCV4

The presence of the PCV3 and the PCV4 genome in the analyzed samples was confirmed with separate real-time PCR assays, which were designed for the study. In the case of PCV3, the modified method described by Krüger et al. (2019) was used [22]. For both PCV3 and PCV4, a Cap gene, encoding capsid protein, was selected as a target region. The reference sequences were retrieved from GenBank: PCV3 was represented by the genomic sequence of CN Nanjing 2017 (GenBank accession no. MK580468), and PCV4 by HNU-AHG1-2019 (GenBank accession no. MK986820) strains. The primers and probes specific for PCV4 were designed in Geneious R9 (BioMatters, Auckland, New Zealand). Synthetic DNA from the Cap gene of both PCVs was used as a positive amplification control. The detailed characteristics of the abovementioned sequences are included in Table 2. The primers and probes were synthetized by Sigma-Aldrich (Burlington, MA, USA). The synthetic DNA of PCV3 was synthetized by Genomed S.A. (Warsaw, Poland) and of PCV4, by Eurofins (Luxembourg City, Luxembourg).

The real-time PCR protocol was optimized using FastStart Universal Probe Master (ROX) (Roche, Basel, Switzerland). A PCR mix was prepared with a final volume of 20 μL, containing 2 μL of the DNA sample, 10 µL of FastStart Universal Probe Master, 0.4 μL of each sense and antisense primer (final concentration 0.4 µM), and 0.2 μL of the probe (final concentration 0.1 µM). The incubation profile for PCV3 and PCV4 DNA amplification was established as follows: 10 min at 95 °C, 40 cycles at 95 °C for 10 s, and 58 °C for 30 s, with fluorescence acquisition in the FAM channel at the end of each PCR cycle. A fluorescent signal in the FAM channel for the analyzed sample was considered positive [22].

In order to exclude potential cross-reactions with other PCVs, the real-time PCR assays for PCV3 and PCV4 detection were performed with the DNA of PCV1 (the strain from the Strains Bank of the Department of Swine Diseases), PCV2, and PCV3 (isolates provided by Dr. Katarzyna Podgórska and Dr. Ewelina Czyżewska-Dors, National Veterinary Research Institute in Pulawy, Poland). Cross-reactions were not observed.

### 2.3. Statistical Analysis and Logistic Regression Models

The statistical analyses were conducted with the application of logistic regression models. This type of model is a mathematical formula that can be used to report the effect of several variables on the dichotomous variable. To obtain the ratings for the regression coefficients, the maximum likelihood method was used. The significance of the independent variables was estimated using the Wald test. The fit of the model to the data was also determined in advance using the likelihood ratio (LR statistics). Odds ratios (ORs) were established with 95% confidence intervals. In all analyses, the significance level α = 0.05 was adopted.

All statistical analyses were conducted with the use of TIBCO Software Inc.’s (Palo Alto, CA, USA) (2017) Statistica (data analysis software system) version 13 (StatSoft Polska, Cracow, Poland), while the map was made in ArcGIS 10.4.1 (Esri Inc., Redlands, CA, USA).

### 2.4. Virus Isolation (PCV3)

Five PCV3-positive samples, showing the lowest Ct value obtained during the real-time PCR, were selected for further virus isolation. For this purpose, two cell lines were applied: PK-15 cells (ATCC CCL-33) (using the method described by Mora-Díaz et al. (2020)) [14] and primary porcine alveolar macrophages (PPAMs) (Walczak et al. (2020), method modified to PCV3 [23]). PK-15 cells were purchased from the ATCC and maintained according to the attached instructions; whereas, PPAMs were acquired from pig lung lavage and maintained as described by Carrascosa et al. (2011) [24].

### 2.5. PCR and Sequencing (PCV3)

Out of the PCV3-positive samples, a total of 10 specimens were subjected to genome sequencing. Since the PCV3 genome is small (approximately 2000 bp, [25]), conventional PCR was used to amplify the sample, before sequencing using the Sanger method. For that purpose, the modified method described by Fux et al. (2018) was used [25]. Three pairs of primers were selected, and one additional pair was designed in Geneious R9 (BioMatters, Auckland, New Zealand). The primers were synthetized by Eurofins (Luxembourg City, Luxembourg). Detailed characteristics of the primers used are included in Table 3.

Amplification was performed using MyTaq HS Red Mix (Meridian Bioscience, Cincinnati, OH, USA), with a total volume of 25 µL, containing: 12.5 µL of Master Mix, 9.5 µL of DNase RNase-free water, 1 µL of each primer with a final concentration of 20 pM, and 1 µL of the DNA template. The cycling protocol was as follows: 1 cycle of 95 °C for 5 min, 30 cycles consisting of denaturation (98 °C for 30 s), annealing (55 or 60 °C for 60 s), and extension (72 °C for 30 s or 2 min). The products were visualized using electrophoresis in 1.8% agarose gels (EurX, Gdańsk, Poland), stained by a SimplySafe dye (EurX, Gdańsk, Poland), diluted according to the manufacturer’s instructions. The molecular weight was traced with a 100 bp DNA Ladder, plus a Gene Ruler (Thermo Fisher Scientific, Waltham, MA, USA). The amplification products were subjected to conventional sequencing using the Sanger method, performed by an outsourcing service, Genomed S.A. (Warsaw, Poland).

### 2.6. Phylogenetic Analysis (PCV3)

The obtained sequences were assembled into full genomes using the Geneious R9 (Biomatters, Auckland, New Zealand) software, and further deposited in the NCBI GenBank database under the following accession numbers: OQ533864–OQ533873.

The obtained whole genome sequences were aligned with each other, as well as with the other 68 PCV3 genomes retrieved from the GenBank database, including the ones originating from Germany, Spain, Russia, Serbia, China, and the USA, using the global alignment algorithm implemented in the Geneious R9 software. The calculated similarity matrix is included in Supplementary Table S1. Based on the obtained alignment, a phylogenetic tree was constructed using MEGA11 software [26]. Detailed information is included below, with each tree presented in Section 3.5.

## 3. Results

### 3.1. Detection of ASFV, PCV3, PCV4

**Figure 1:**
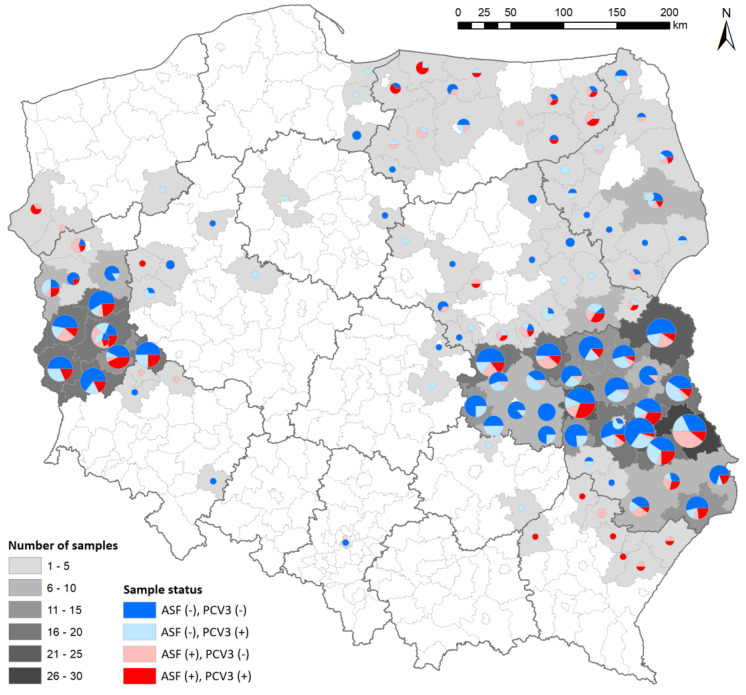
The distribution of the analyzed samples in Poland (map divided into voivodships).

The ASF status declared by the NRL for the ASF of the analyzed samples was confirmed. There were 480 ASFV-negative (group I) and 200 ASFV-positive (group II) samples, including 143 molecularly positive (real-time PCR) and 57 serologically positive (IPT) specimens. All the samples were further analyzed for the presence of PCV3 and PCV4 genetic material. None of samples showed the presence of PCV4. When it came to PCV3, its DNA was detected in 257 samples, representing 37.8% (32.1–41.6%) of the analyzed samples from 11 voivodships. In regard to group I (ASFV−), PCV3 genetic material was detected in 154 samples (32.1% (27.9–36.5%)) from 10 Polish voivodships. In regard to group II (ASFV+), PCV3 genetic material was detected in 103 samples (51.5% (44.3–58.6%)) from seven Polish voivodships. The distribution of the analyzed samples, with their ASF and PCV3 status, is presented in Figure 1.

The oldest sample in which the PCV3 genome was detected came from a wild boar that was found dead, in which the presence of ASFV genetic material was also confirmed. The animal was found on January 31, 2018, in the Warmińsko-Mazurskie voivodship (Braniewo county, Lelkowo municipality, a forest area). The ASFV and PCV3 status concerning the year and location are presented in Table 4.

Analysis of the samples from groups I and II showed no presence of PCV4 genetic material.

### 3.2. Logistic Regression Models

All the analyzed logistic regression models examined the influence of various variables on the occurrence of the PCV3 virus in the sample. Variables, such as the ASFV score, year, and quarter were significant (*p* < 0.0001) in the selected the best-fit model.

In addition to the positive impact of the ASF result on the PCV3 result, the model also showed a significant impact of the quarter, specifically the positive impact of the second and fourth quarters compared to the first, and the year, specifically 2018 compared to 2019.

The odds of a PCV3(+) result for ASF(+) was more than two times higher (OR = 2.3 (95% CI: 1.6–3.4)) than for ASF(−) (*p* < 0.0001).

The odds of a PCV3(+) result in 2018 was almost three times higher (OR = 3.0 (95% CI: 1.7–5.1)) than in 2019 (*p* = 0.0001). The years 2020 and 2021 did not differ significantly from 2019, when the percentage of PCV3(+) results was the lowest.

The odds of a PCV3(+) result in Q2 was more than 1.5 times higher (OR = 1.6 (95% CI: 1.1–2.4)) than in Q1 (*p* = 0.01) and almost twice as high (OR = 1.9 (95% CI: 1.1–3.2)) in Q4 than in Q1 (*p* = 0.02). Q3 did not differ significantly from Q1, in which PCV3 prevalence was the lowest.

Voivodships did not show a significant impact on the PCV3 result. Only in one model, which included five selected voivodships (645 samples out of 680) and the year and the ASF status, the Lubuskie voivodship indicated a significant positive impact on the PCV3(+) result, which had a more than 2.5 times greater chance than for the Podlaskie voivodship. However, when additional quarters were taken into account in the modelling, this dependence became insignificant.

### 3.3. Virus Isolation

Five tissue homogenates (three lungs, one spleen, one tonsil) were selected for the isolation of PCV3, of which three belonged to group I (ASFV−) and two to group II (ASFV+). The samples showing the lowest Ct values in the real-time PCR test (range from 23.87 to 24.86) were selected due to the largest potential number of virus copies in the analyzed sample. After inoculation, the microscopic observations were conducted for three days, then the samples were further passaged three times in the same manner, during which real-time PCR was performed to monitor the virus replication. However, a cytopathic effect was not observed, suggesting that the virus was not replicating in PK-15 cells, which was confirmed with the real-time PCR test. In summary, the isolation of a viable PCV3 virus on the PK-15 cell line was unsuccessful.

Similarly, the same samples were used in the trial of virus isolation in PPAMs. After inoculation, the microscopic observations were conducted for eight days (after four days the culture medium was replaced), during which real-time PCR was performed to monitor the virus replication. As in the case of PK-15, a cytopathic effect was not observed, suggesting that the virus was not replicating in PPAM cells, which was also confirmed with the real-time PCR test. The isolation of a viable PCV3 virus on the PPAM cells was unsuccessful.

### 3.4. Conventional PCR and Genome Sequencing (PCV3)

For the PCV3 genome amplification, ten samples (lung, spleen, tonsil, or blood) from five voivodships (Lubelskie, Lubuskie, Mazowieckie, Podlaskie, Podkarpackie) were selected, including three from group I (ASFV−) and seven from group II (ASFV+). As in the case of virus isolation, the samples with the lowest Ct values (range from 23.86 to 26.33) were selected for the PCR test and sequencing. All the PCR products showed correct sizes; thus, they were further subjected to sequencing. After sequencing, 10 complete PCV3 genome sequences were assembled. The genes were annotated using Geneious R9, and the genomes were deposited in the GenBank database. The obtained genomes showed a typical length of about 2000 bp (range from 1999 to 2004 bp). Detailed data associated with the samples are presented in Table 5.

### 3.5. Phylogenetic Analysis (PCV3)

**Figure 2:**
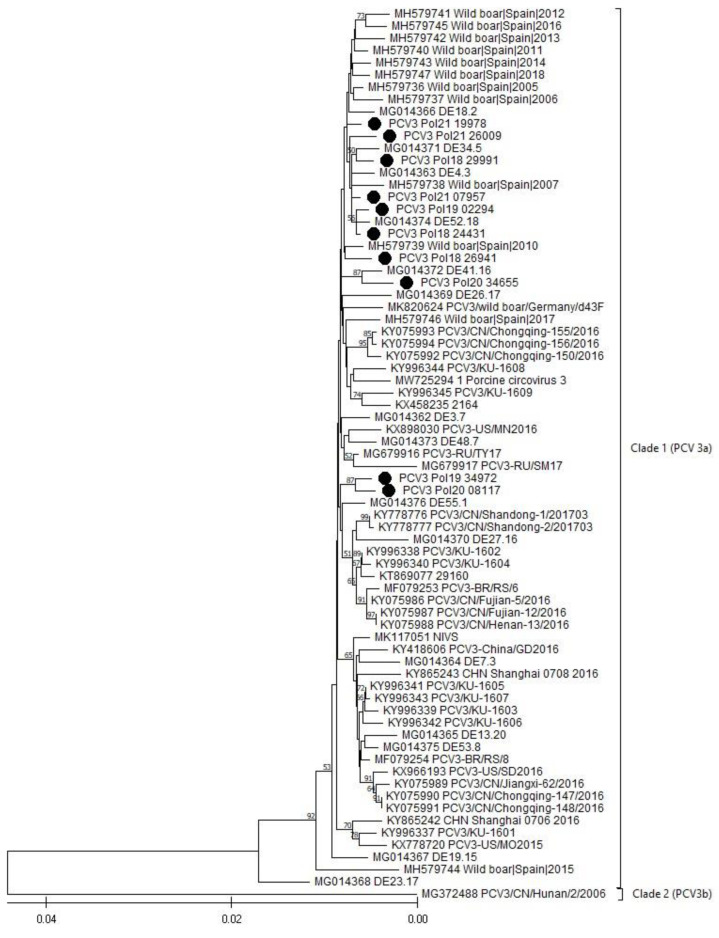
The phylogenetic tree is based on PCV3 complete genome sequences. The sequences generated in this study are designated with a black dot (•). The evolutionary history was inferred using the neighbor-joining method [27]. The optimal tree is shown. The percentage of replicate trees, in which the associated taxa clustered together in the bootstrap test (1000 replicates), is shown above the branches [28]. The tree is drawn to scale, with branch lengths in the same units as those of the evolutionary distance used to infer the phylogenetic tree. The evolutionary distances were computed using the maximum composite likelihood method [29], and are in the units of the number of base substitutions per site.

**Figure 3:**
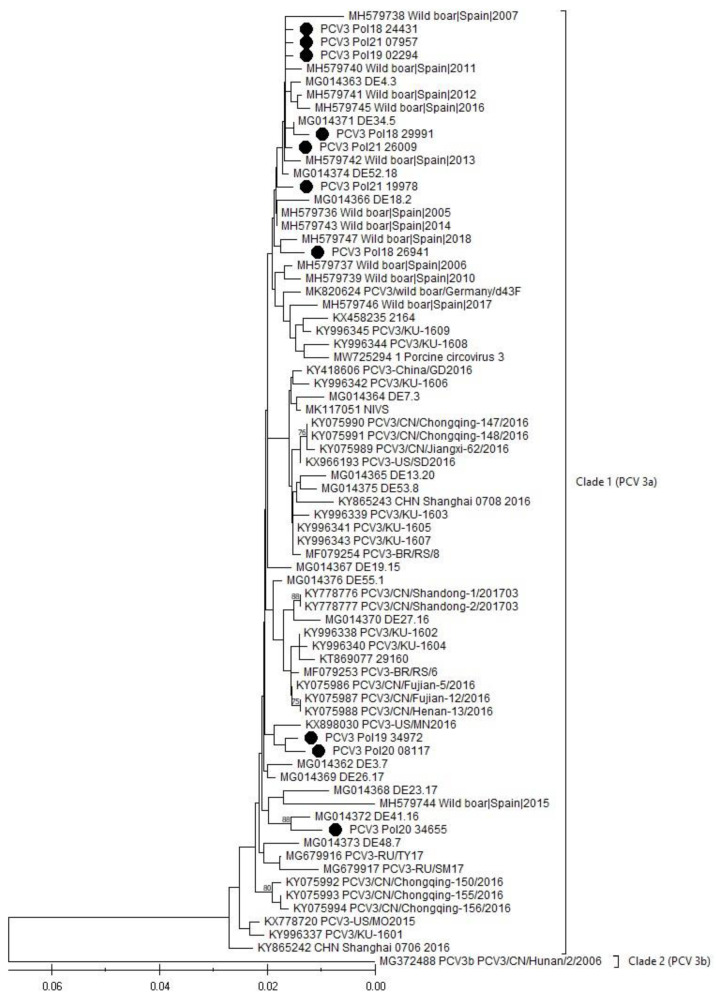
The phylogenetic tree based on the Cap gene sequence of PCV3. The sequences generated in this study are designated with a black dot (•). The evolutionary history was inferred using the neighbor-joining method [27]. The optimal tree is shown. The percentage of replicate trees, in which the associated taxa clustered together in the bootstrap test (1000 replicates), is shown above the branches [28]. The tree is drawn to scale, with branch lengths in the same units as those of the evolutionary distance used to infer the phylogenetic tree. The evolutionary distances were computed using the maximum composite likelihood method [29], and are in the units of the number of base substitutions per site.

The phylogenetic analysis, performed based on a full genome (Figure 2) and a capsid gene (Figure 3) of the obtained PCV3 viruses, showed that all sequences belonged to Clade I (PCV3a), the most prevalent in the world (Figure 2 and Figure 3).

The alignment of the 10 Polish PCV3 whole genomes showed high sequence similarity within the range of 98.78% to 99.80% (Supplementary Table S1). The similarity between the Polish PCV3 wild boar strain and the neighboring German PCV3 wild boar strain ranged from 98.952 to 99.35 (Supplementary Table S1). In regard to the other 68 PCV3 genomes selected from the GenBank, the similarity ranged from 90.84% to 99.80% (Supplementary Table S1). However, excluding the only representative of the PCV3b clade, the sequence similarity between the Polish and the other PCV3a strains oscillates between 98.03% and 99.80%.

## 4. Discussion

The ASF epidemic in Poland has been ongoing since 2014, and the perspectives on disease eradication are still low [30]. The disease causes enormous economic losses [31] and leads to a decrease in the number of pig farms in Poland [32]. The alarming fact is that the number of ASF outbreaks both in domestic pigs and wild boars is constantly at a high level (2021: 124 ASF outbreaks in domestic pigs, 3214 ASF outbreaks in wild boars) [32]. Moreover, recent data suggest that the disease is endemic in the wild boar population [33].

PCV-associated syndromes, such as PCV2 systemic disease (PCV2-SD), reproductive disease, and porcine dermatitis and nephropathy syndrome (PDNS) are well known and are comprehensively described concerning PCV2 infection [34]. However, the pathogenesis of PCV3 is poorly documented, and there is even less information about PCV4 clinical outcomes [12]. The data collected from pig farms in the United States of America (USA) indicate that PCV3 may cause reproductive failures, encephalitis and myocarditis in perinatal piglets, periarteritis and, similar to PCV2, PDNS [35].

PCV3 was detected for the first time in Polish domestic pigs in 2017. The virus was confirmed in 12 out of 14 analyzed farms. PCV3 was detected in asymptomatic pigs, as well as in animals with PCVD [5]. In addition to these data, we have confirmed that PCV3 is also widespread in the wild boar population in Poland, similar to other European countries.

It is well known that coinfections lead to an increase in incidence and a more severe clinical course of PCVD caused by PCV2 [36,37,38,39]. The data from the USA indicate that 98% of PCVAD cases occurred in the form of coinfection [39]. Coinfection may occur with other viruses (i.e., porcine parvovirus, porcine reproductive and respiratory syndrome virus (PRRSV), pseudorabies virus, classical swine fever virus, porcine epidemic diarrhea virus (PEDV), swine influenza virus) [37,38], bacteria (i.e., *Mycoplasma hyopneumoniae*, *Salmonella* spp.) [36,38], or even other PCVs [38]. Recent data indicate that not only PCV2, but also PCV3, may coinfect domestic pigs with ASFV [19,20,21]. PCV3 coinfection was also confirmed with PEDV [40].

In this study, coinfection with PCV3 and ASFV in the wild boar population has been confirmed for the first time. Moreover, we showed that ASFV infection has significantly higher odds in PCV3-infected individuals, compared to PCV3-free animals (OR = 2.3 (95% CI: 1.6–3.4)). This finding complements earlier reports showing that coinfection with PCVs may favor the development or enhance the course of the other diseases [36]. Swine circoviruses have tropism to the lymphoid tissue of the host, thereby leading to immunosuppression and an increased tendency to secondary infections [41]. Moreover, it has been demonstrated that PCV2 and PRRSV coinfection promoted PRRSV replication in PAM cells. The downregulation of immune regulatory factors (IFN-α and IFN-γ) and the upregulation of inflammatory factors (TNF-α, IL-1β, IL-10, and TGF-β) and immune checkpoint molecules (PD-1, LAG-3, CTLA-4, and TIM-3) were observed in the cells where PRRSV infection was preceded by PCV2 [41]. Consistent with these reports, our results suggest that primary PCV3 infection, similar to PCV2, may play a role in susceptibility to the other viruses, including ASFV. Obviously, this proposition needs further investigation. These data may be crucial in regard to ASF epidemiology and eradication.

PCV3 isolation in established cell lines is a tough task, since to date only a few reports have confirmed its successful isolation with the use of primary porcine kidney cells [42] and with the continuous PK-15 cell line [14]. However, in both cases, the virus was isolated from fresh samples, including young domestic pigs, such as clinically healthy 91-day-old [42], 1-day-old, and 8-day-old piglets, or fetuses (mummified and still born) [14]. The samples contained a high viral load, which was illustrated by a very low Ct level, in the range between 7.5 and 19.3, obtained during real-time PCR tests [14]. Unfortunately, our attempts to isolate PCV3 in the PK-15 cell line and primary porcine macrophages (PPAMs) failed. In contrast to Oh and Chae (2020) [42] and Mora- Díaz et al. 2020 [14], our samples were collected from adult wild boar carcasses and road killed animals delivered from the field during the ASF monitoring program in Poland. The time they spent in the field was unknown. Since viruses are susceptible to unfavorable conditions, it may be hypothesized that PCV3 virions were damaged during the time spent in the environment. Despite the fact that the blood samples were freshly collected from hunted animals, the viral load was likely too low to facilitate successful virus isolation. This hypothesis was reflected by a real-time PCR test, where relatively high Ct levels for PCV3 were observed, and the lowest Ct obtained was 23.86, much higher than documented by Mora-Díaz et al. (2020) [14].

PCV3 is one of the smallest known viruses. The genome size is about 2000 bp and the similarity between its known sequences is in the range of 97–100% [25,43]. The most recent comprehensive phylogenetic analysis, conducted by Franzo et al. (2020), indicated that all PCV3 strains may be divided into two clades, PCV3a and PCV3b; however, in the GenBank database, only one representative of the 3b clade is currently deposited, raising questions about its authenticity [44]. As expected, all PCV3 genomes obtained during our study showed a high level of nucleotide similarity, within the range from 98.78% to 99.80%. The neighbor-joining analysis, integrating 68 other isolates selected from around the world, showed that the generated sequences belong to the most abundant PCV3a genotype, and also show a very high level of sequence similarity with other members of the 3a clade (range between 98.03 and 99.80%). The minor differences were identified as silent mutations, or changes within noncoding regions. Their influence on the virus characteristics and disease outcomes are unknown.

PCV3 has been confirmed both in domestic pigs and in wild boars in Europe; however, this is the very first report showing the presence of PCV3 in wild boars in Poland. Moreover, we are the first to document PCV3 coinfection with ASFV in wild boars in Poland. The odds of PCV3 infection were about 2.3 times higher in ASFV-infected individuals, suggesting that the circovirus carrier state may increase the chances of developing ASFV. The knowledge about both viruses, their synergy and prevalence in wild boars is crucial to estimate the risk for domestic pigs and for maintaining the safety of Polish swine herds.

## 5. Conclusions

In the current study, we demonstrated the high prevalence of PCV3 in the wild boar population in Poland; meanwhile, PCV4 was not detected. The odds of ASFV infection on the grounds of the PCV3 carrier state in free-ranging Suidae in Poland was more than twice as high than in the group of PCV3-free animals. Ten full genome sequences of PCV3 were obtained, all of them belonging to clade 3a. The similarity between them was in the range 98.78–99.80%.

## Figures and Tables

**Table 1:** Characteristics of samples used in the study.

Voivodship	Group I (ASFV−)	Group II (ASFV+)	Total
Dolnośląskie	2	2	4
Kujawsko-Pomorskie	2	0	2
Lubelskie	229	73	302
Lubuskie	82	49	131
Łódzkie	1	0	1
Mazowieckie	112	30	142
Podkarpackie	0	10	10
Podlaskie	24	9	33
Pomorskie	3	0	3
Śląskie	1	0	1
Świętokrzyskie	2	0	2
Warmińsko-Mazurskie	14	23	37
Wielkopolskie	7	0	7
Zachodniopomorskie	1	4	5
**Total**	**480**	**200**	**680**

**Table 2:** Primers, probes, and controls designed for PCV3 and PCV4.

PCR Target	Name	Type	Sequence (5′-3′)	Nucleotide Position **	Length [bp]
PCV3 Cap	PCV3-Cap-F *	primer	AGTGCTCCCCATTGAACG	1434–1451	18
	PCV3-Cap-R *	primer	ACACAGCCGTTACTTCAC	1551–1568	18
	PCV3-probe *	probe	6FAM-ACCCCATGGCTCAACACATATGACC-BHQ1	1456–1480	25
	PCV3-control	synthetic DNA	AGT GCT CCC CAT TGA ACG GTG GGG TCA TAT GTG TTG AGC CAT GGG GTG GGT CTG GAG GAG AAA AAG AGG CTT TGT CCT GGG TGA GCG CTG GTA GTT CCC GCC AGA ATT GGT TTG GGG GTG AAG TAA CGG CTG TGT	1434–1568	135
PCV4 Cap	PCV4-Cap-F	primer	AGGTTTACGATCGTTCCC	1486–1503	18
	PCV4-Cap-R	primer	CTTCATGAGGGAGGTGAC	1588–1605	18
	PCV4-probe	probe	6FAM-TAATGTCCAACGTTCCAAGAGGGCG-BHQ1	1540–1564	25
	PCV4-control	synthetic DNA	CTG TAA AGG TTT ACG ATC GTT CCC GGT CCT TTT GGG ATA AAG TCC TTC AGT TTG AAA TCG TAA TGT CCA ACG TTC CAA GAG GGC GTG GAA AAG CTT GAC ACG CTG AGA GTC ACC TCC CTC ATG AAG CGC GCAT	1492–1624	133

* Primers and probes described by Krüger et al. 2019 [22]; ** in reference to MK580468 for PCV3 and MK986820 for PCV4.

**Table 3:** Primers used for amplification and sequencing of PCV3 genome fragments.

Primer	Sequence (5′-3′)	Length of Amplified Sequence [bp]	Annealing Temperature [°C]	Extension Time [s]
PCV3_74_ F *	CACCGTGTCAGTGGATATAC	1072	55	120
PCV3_1144_ R *	CACCCCAACGCAATAATTGTA			
PCV3_1137_ F *	TTGGGGTGGGGGTATTTATT	425	55	120
PCV3_1561_ R *	ACACAGCCGTTACTTCAC			
PCV3_1427_ F *	AGTGCTCCCCATTGAACG	1007	55	120
PCV3_433_ R *	CGACCAAATCCGGGTAAGC			
PCV3_911_ F	GGGTTCCTGTTAAGGGTGGG	487	60	30
PCV3_1415_ R	GACAGACTTCTACGGCACCA			

* Primers described by Fux et al., 2018 [25].

**Table 4:** The distribution of the results (year/voivodship).

Year/Voivodship	Group I-ASFV (−)			Group II-ASFV (+)			Total
	PCV3 (−)	PCV3 (+)	Total	PCV3 (−)	PCV3 (+)	Total	
2018	35	45	80	14	8	22	102
2019	115	45	160	27	21	48	208
2020	120	40	160	21	29	50	210
2021	56	24	80	35	45	80	160
Dolnośląskie	2	0	2	2	0	2	4
Kujawsko-Pomorskie	1	1	2	0	0	0	2
Lubelskie	161	68	229	36	37	73	302
Lubuskie	58	24	82	17	32	49	131
Łódzkie	1	0	1	0	0	0	1
Mazowieckie	74	38	112	18	12	30	142
Podkarpackie	0	0	0	4	6	10	10
Podlaskie	14	10	24	7	2	9	33
Pomorskie	2	1	3	0	0	0	3
Śląskie	1	0	1	0	0	0	1
Świętokrzyskie	0	2	2	0	0	0	2
Warmińsko-Mazurskie	8	6	14	11	12	23	37
Wielkopolskie	4	3	7	0	0	0	7
Zachodniopomorskie	0	1	1	2	2	4	5
**Total**	**326**	**154**	**480**	**97**	**103**	**200**	**680**

**Table 5:** Characteristics of the obtained PCV3 genomes.

Name	Group	Year	Origin	Sample Type	Sample Ct (PCV3)	Voivodship	GenBank Accession No.
PCV3_Pol18_24431	I	2018	road killed	lung	24.11	Mazowieckie	OQ533868
PCV3_Pol18_26941	I	2018	found dead	spleen	23.87	Podlaskie	OQ533864
PCV3_Pol18_29991	I	2018	found dead	tonsil	24.72	Lubelskie	OQ533871
PCV3_Pol19_02294	II	2019	found dead	lung	24.86	Lubelskie	OQ533869
PCV3_Pol19_34972	II	2019	found dead	lung	23.99	Lubelskie	OQ533865
PCV3_Pol20_08117	II	2020	road killed	spleen	23.86	Lubuskie	OQ533866
PCV3_Pol20_34655	II	2020	found dead	lung	25.17	Lubuskie	OQ533872
PCV3_Pol21_07957	II	2021	found dead	spleen	26.33	Podkarpackie	OQ533870
PCV3_Pol21_19978	II	2021	found dead	spleen	25.27	Lubuskie	OQ533873
PCV3_Pol21_26009	II	2021	hunted	blood	25.70	Lubelskie	OQ533867

## Data Availability

Whole genome sequences of PCV3s in the project are available from GenBank under accession no. OQ533868, OQ533864, OQ533871, OQ533869, OQ533865, OQ533866, OQ533872, OQ533870, OQ533873, OQ533867.

## Supplementary Materials

The following supporting information can be downloaded at: https://www.mdpi.com/article/10.3390/v16050754/s1, Table S1: The alignment of PCV3 whole genomes.
